# Chronically jailed right ventricular implantable cardioverter defibrillator lead visualized with imaging integration prior to ventricular tachycardia ablation

**DOI:** 10.1007/s10840-025-02054-3

**Published:** 2025-05-14

**Authors:** Nithi Tokavanich, Jackson Liang

**Affiliations:** Section of Cardiac Electrophysiology, University of Michigan Medical Center, SPC 5853, 1500 E. Medical Center Drive Cardiovascular Center, Ann Arbor, MI 48109 - 5853 USA

A 73-year-old man with ischemic cardiomyopathy and ventricular tachycardia (VT) previously underwent implant of single-chamber implantable cardioverter defibrillator (ICD) in 2012. In 2016, following multiple strokes he underwent patent foramen ovale (PFO) closure with an Amplatzer Talisman PFO closure device (Abbott, Chicago, IL) at an outside facility. Several years later, the experienced multiple ICD shocks due to sustained monomorphic VT, and was referred to our institute for VT ablation.

**Fig. 1:**
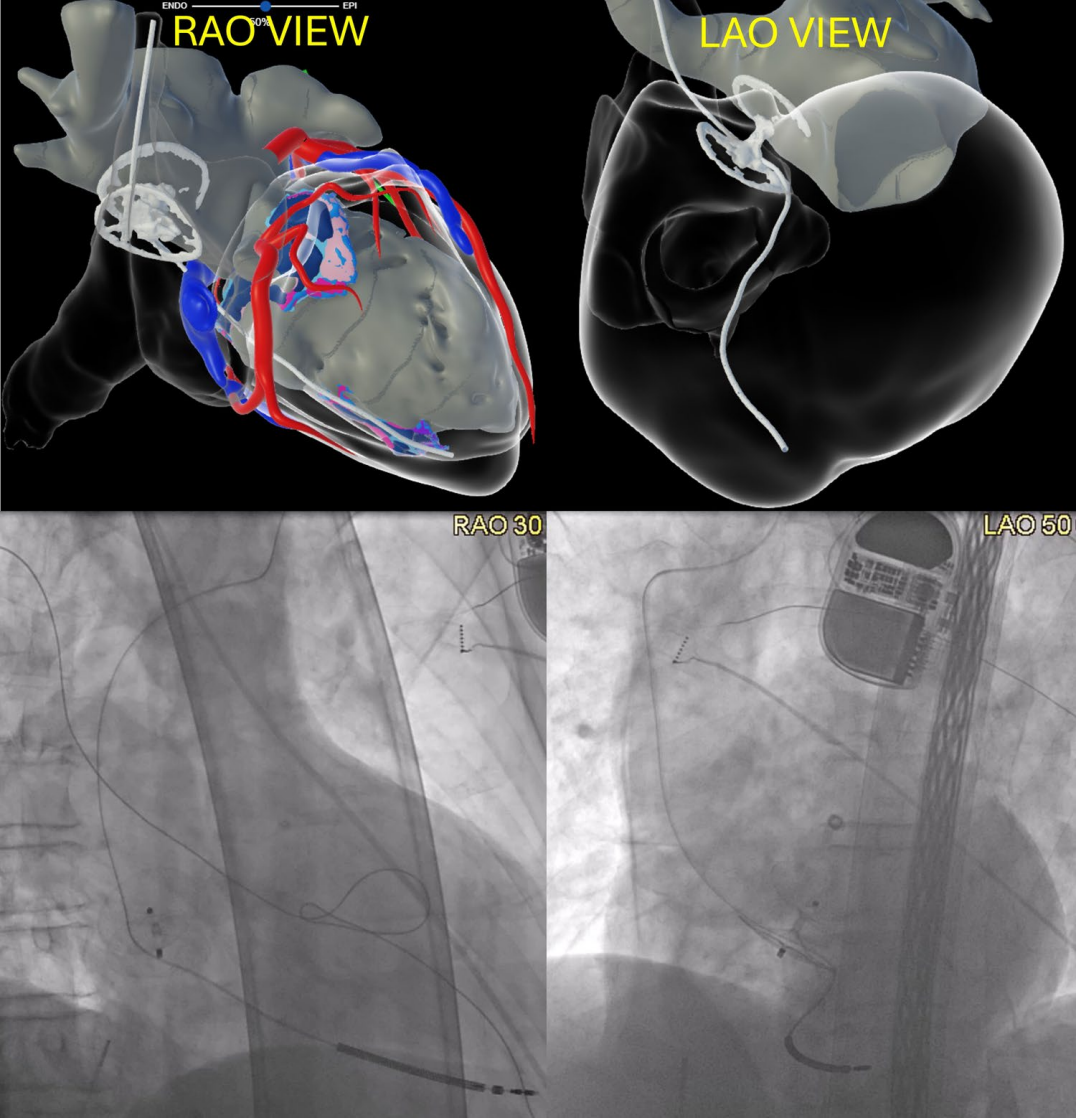
InHEART images (RAO and LAO views, top panel) and fluoroscopic images (RAO and LAO views, bottom panel) showing RV ICD lead entrapped within the Amplatzer Talisman PFO closure device

Cardiac magnetic resonance imaging (MRI) and cardiac computed tomography (CT) were performed for pre-procedure planning. Image integration (InHEART, Pessac, France) incidentally demonstrated that the RV ICD lead had been jailed between the PFO closure device and the interatrial septum (Fig. 1, top), which had previously occurred during deployment of the PFO closure device years ago. The RV lead parameters were stable (sensing 6.1 mV, pacing impedance 424 ohms, shock lead impedance 66 ohms, pacing threshold of 0.6 V at 0.4 ms) so the decision was made to continue with conservative management for the lead. He underwent successful VT ablation and eventually underwent upgrade to cardiac resynchronization therapy device (addition of right atrial and left bundle branch area pacing leads).

Given the stable lead function and the risks associated with lead and device extraction, we opted for conservative management with close monitoring of lead parameters every three months.

The InHEART platform constructs a 3D cardiac model by integrating cardiac CT and MRI images, providing detailed anatomical and tissue visualization. While this imaging technique is typically utilized for VT ablation planning, its utility to detect lead-related complications has not been previously described [1].

Lead entrapment is a rare complication of PFO closure. To date, only two cases have been reported, both of which were identified during the PFO device deployment, allowing lead removal before full deployment [2, 3]. Our case represents the first documented instance of chronic RV lead entrapment by a PFO closure device which was incidentally identified with imaging integration prior to VT ablation.
